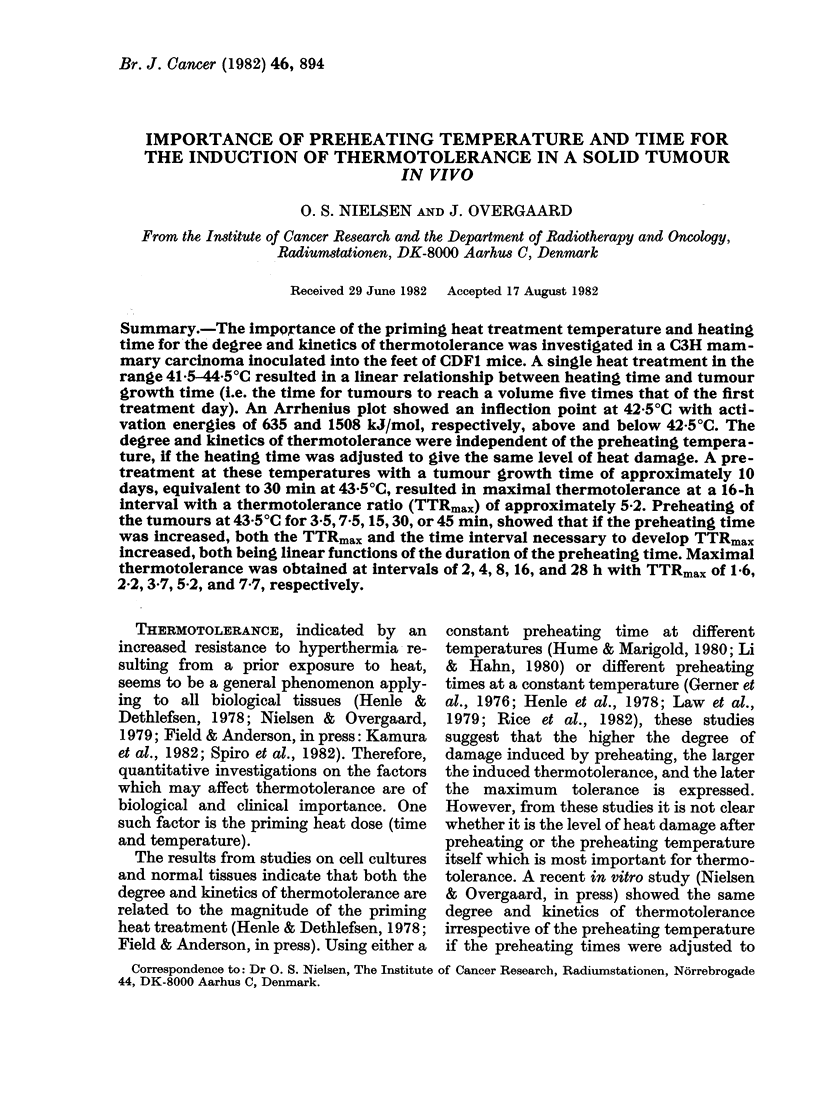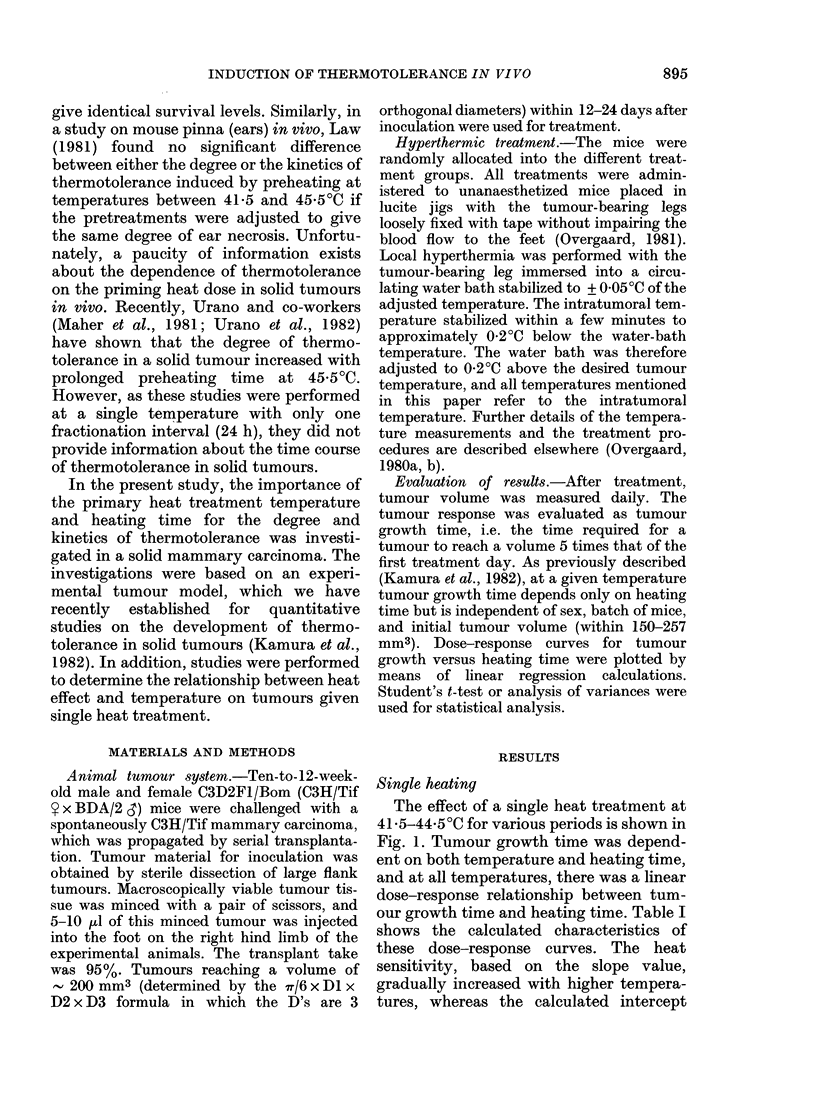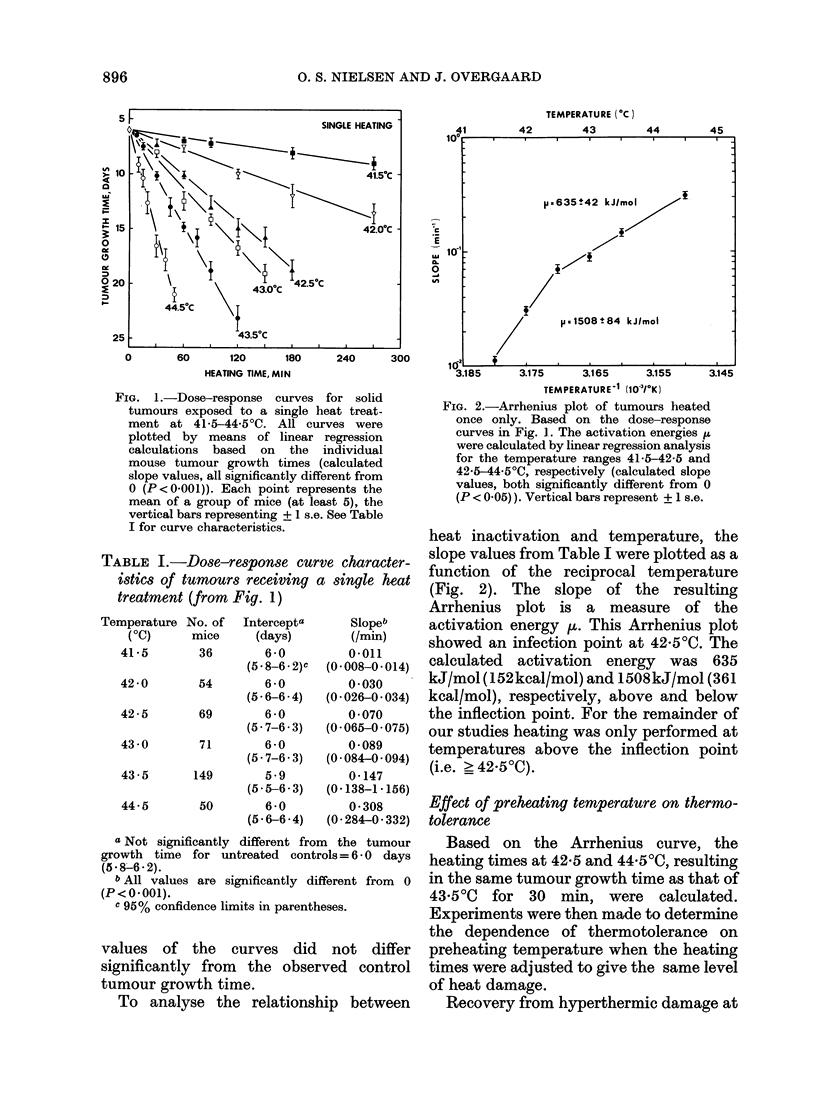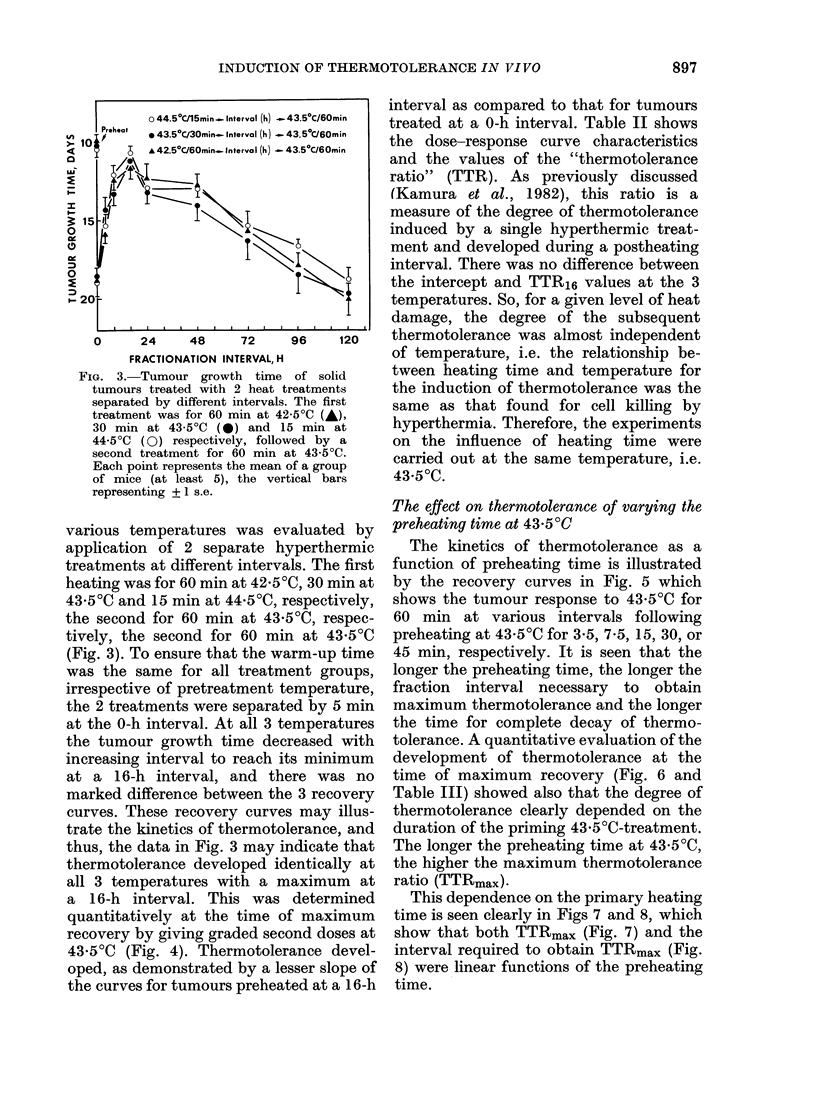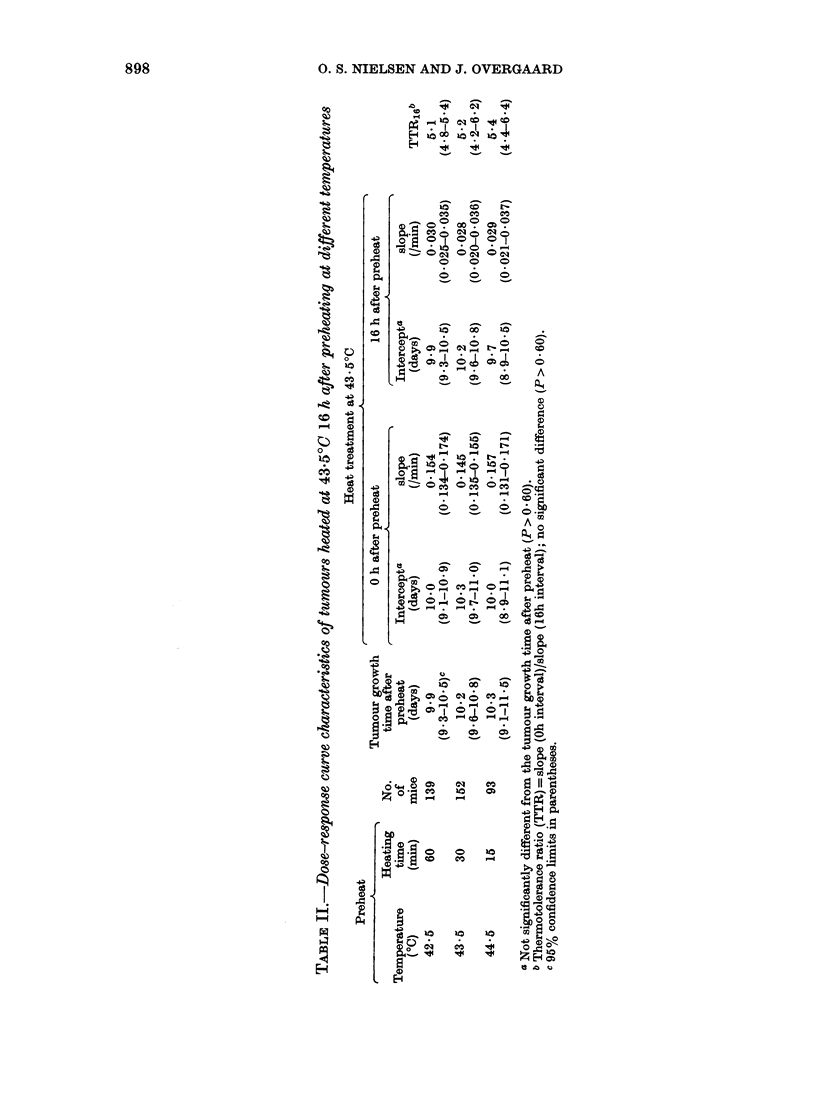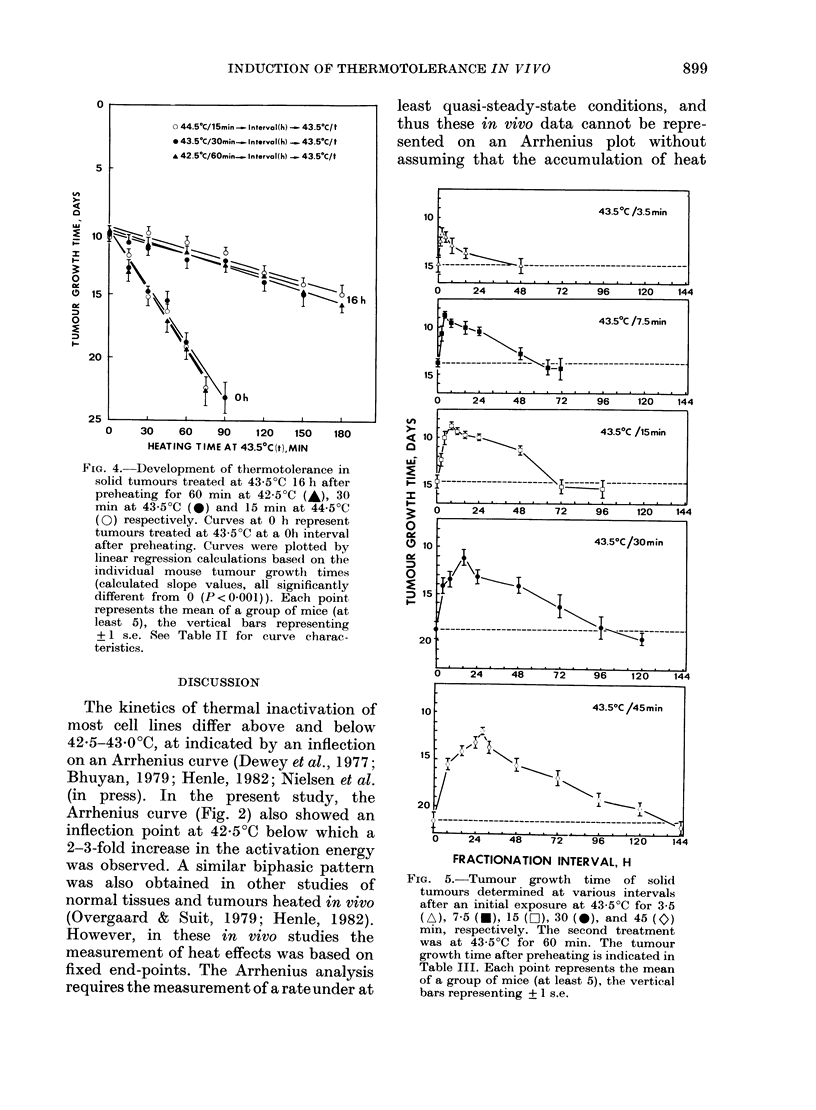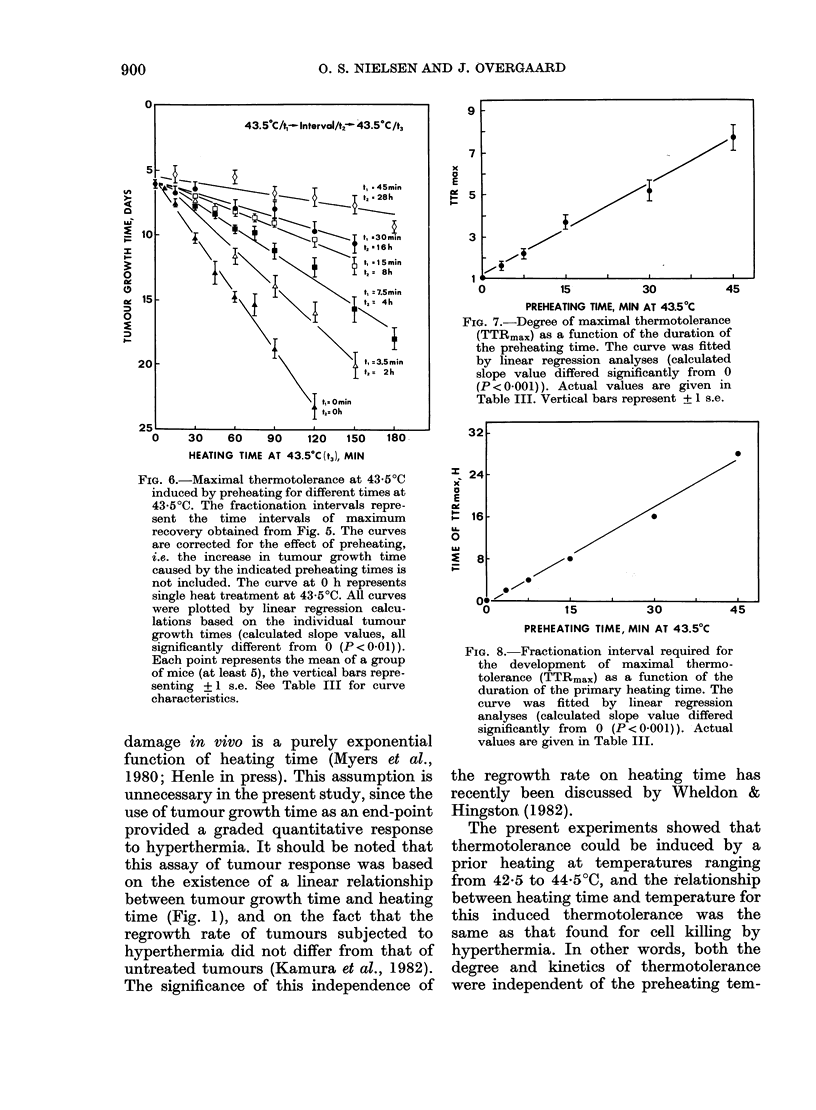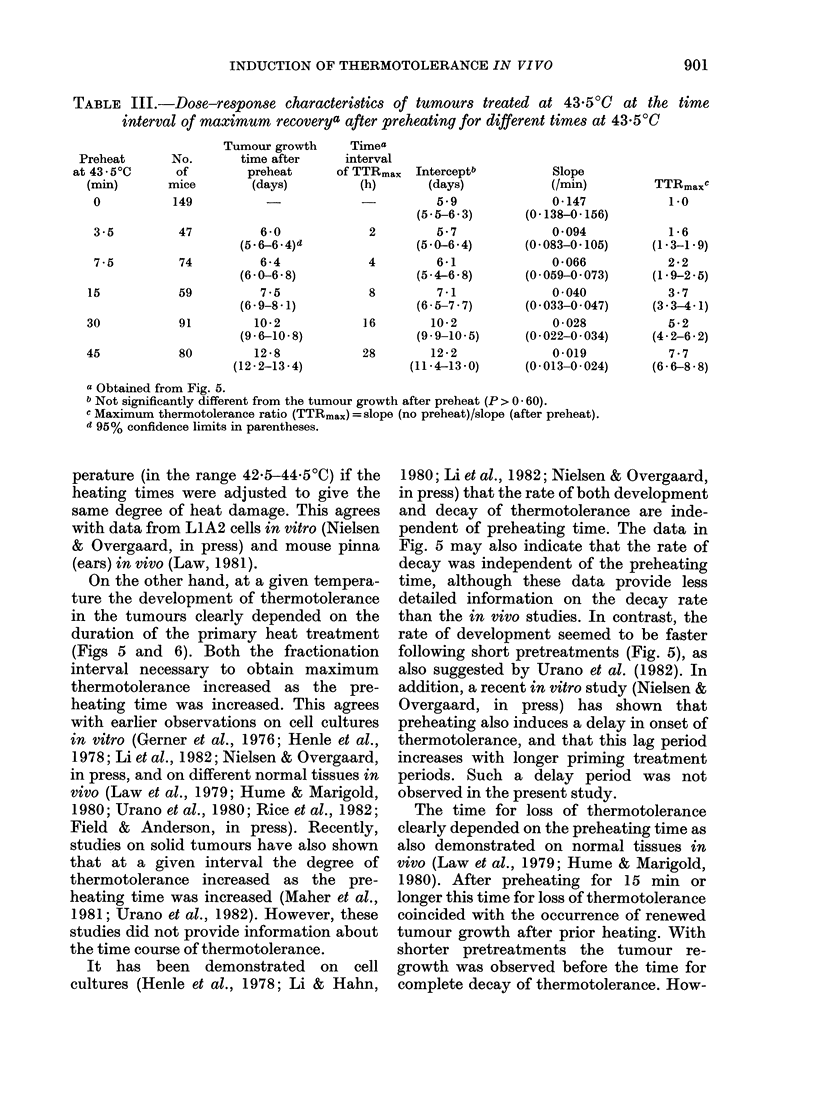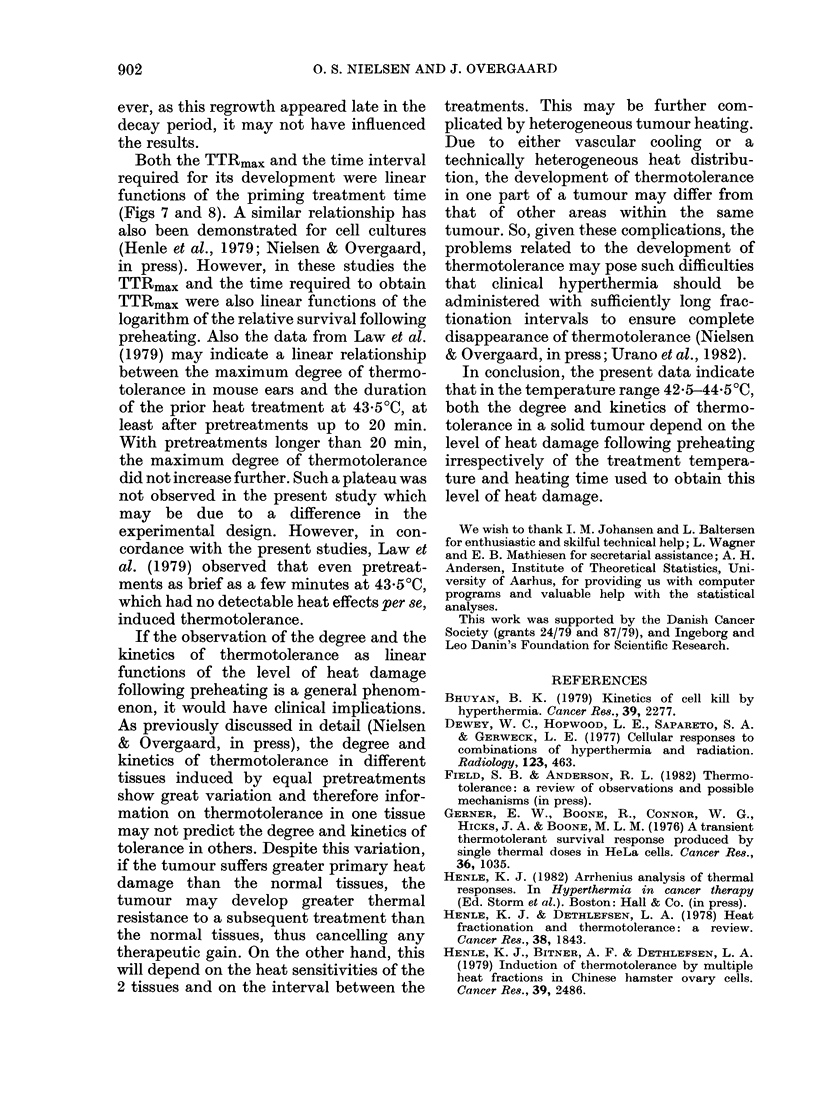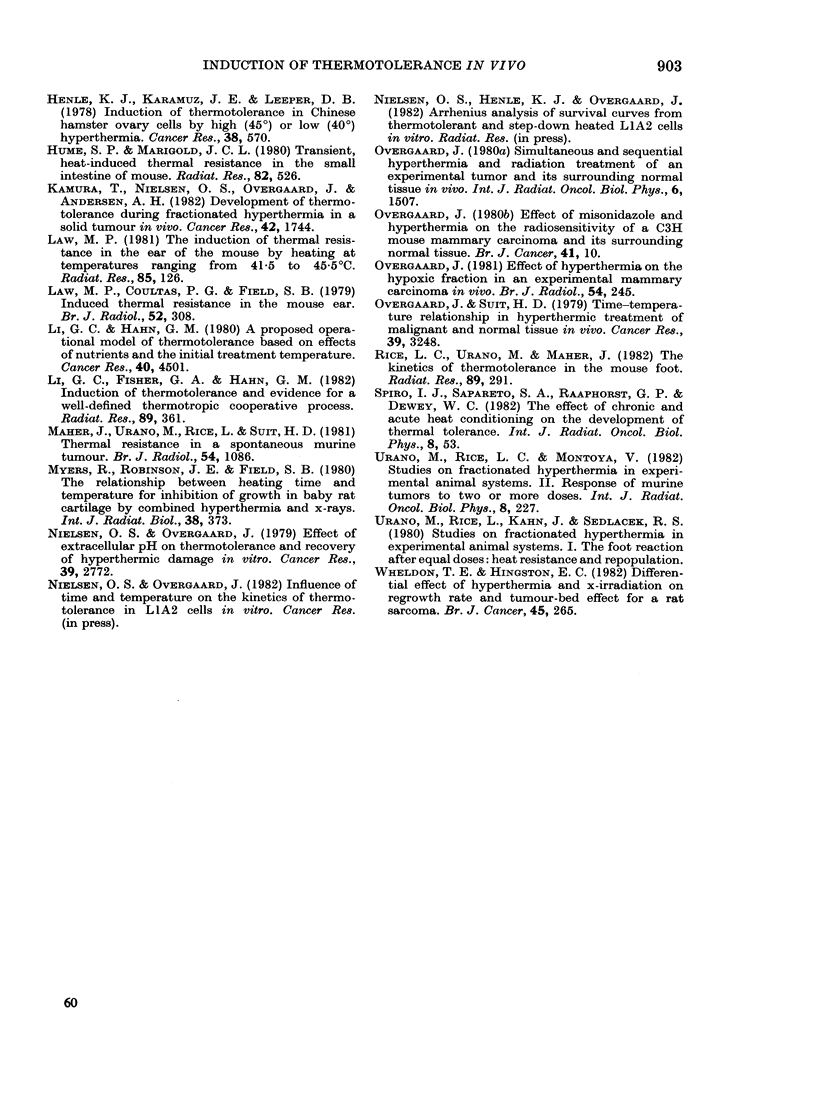# Importance of preheating temperature and time for the induction of thermotolerance in a solid tumour in vivo.

**DOI:** 10.1038/bjc.1982.299

**Published:** 1982-12

**Authors:** O. S. Nielsen, J. Overgaard

## Abstract

The importance of the priming heat treatment temperature and heating time for the degree and kinetics of thermotolerance was investigated in a C3H mammary carcinoma inoculated into the feet of CDF1 mice. A single heat treatment in the range 41.5-44.5 degrees C resulted in a linear relationship between heating time and tumour growth time (i.e. the time for tumours to reach a volume five times that of the first treatment day). An Arrhenius plot showed an inflection point at 42.5 degrees C with activation energies of 635 and 1508 kJ/mol, respectively, above and below 42.5 degrees C. The degree and kinetics of thermotolerance were independent of the preheating temperature, if the heating time was adjusted to give the same level of heat damage. A pretreatment at these temperatures with a tumour growth time of approximately 10 days, equivalent to 30 min at 43.5 degrees C, resulted in maximal thermotolerance at a 16-h interval with a thermotolerance ratio (TTRmax) of approximately 5.2. Preheating of the tumours at 43.5 degrees C for 3.5, 7.5, 15, 30, or 45 min, showed that if the preheating time was increased, both the TTRmax and the time interval necessary to develop TTRmax increased, both being linear functions of the duration of the preheating time. Maximal thermotolerance was obtained at intervals of 2, 4, 8, 16, and 28 h with TTRmax of 1.6, 2.2, 3.7, 5.2, and 7.7, respectively.


*Br. J. Cancer* (1982) **46**, 894

### IMPORTANCE OF PREHEATING TEMPERATURE AND TIME FOR THE INDUCTION OF THERMOTOLERANCE IN A SOLID TUMOUR *IN VIVO*

O. S. NIELSEN AND J. OVERGAARD

*From the Institute of Cancer Research and the Department of Radiotherapy and Oncology, Radiumstationen, DK-8000 Aarhus C, Denmark*

Received 29 June 1982 Accepted 17 August 1982

**Summary.—The importance of the priming heat treatment temperature and heating time for the degree and kinetics of thermotolerance was investigated in a C3H mammary carcinoma inoculated into the feet of CDF1 mice. A single heat treatment in the range 41·5–44·5°C resulted in a linear relationship between heating time and tumour growth time (i.e. the time for tumours to reach a volume five times that of the first treatment day). An Arrhenius plot showed an inflection point at 42·5°C with activation energies of 635 and 1508 kJ/mol, respectively, above and below 42·5°C. The degree and kinetics of thermotolerance were independent of the preheating temperature, if the heating time was adjusted to give the same level of heat damage. A pretreatment at these temperatures with a tumour growth time of approximately 10 days, equivalent to 30 min at 43·5°C, resulted in maximal thermotolerance at a 16-h interval with a thermotolerance ratio ($TTR_{max}$) of approximately 5·2. Preheating of the tumours at 43·5°C for 3·5, 7·5, 15, 30, or 45 min, showed that if the preheating time was increased, both the $TTR_{max}$ and the time interval necessary to develop $TTR_{max}$ increased, both being linear functions of the duration of the preheating time. Maximal thermotolerance was obtained at intervals of 2, 4, 8, 16, and 28 h with $TTR_{max}$ of 1·6, 2·2, 3·7, 5·2, and 7·7, respectively.**

THERMOTOLERANCE, indicated by an increased resistance to hyperthermia resulting from a prior exposure to heat, seems to be a general phenomenon applying to all biological tissues (Henle & Dethlefsen, 1978; Nielsen & Overgaard, 1979; Field & Anderson, in press: Kamura *et al.*, 1982; Spiro *et al.*, 1982). Therefore, quantitative investigations on the factors which may affect thermotolerance are of biological and clinical importance. One such factor is the priming heat dose (time and temperature).

The results from studies on cell cultures and normal tissues indicate that both the degree and kinetics of thermotolerance are related to the magnitude of the priming heat treatment (Henle & Dethlefsen, 1978; Field & Anderson, in press). Using either a constant preheating time at different temperatures (Hume & Marigold, 1980; Li & Hahn, 1980) or different preheating times at a constant temperature (Gerner *et al.*, 1976; Henle *et al.*, 1978; Law *et al.*, 1979; Rice *et al.*, 1982), these studies suggest that the higher the degree of damage induced by preheating, the larger the induced thermotolerance, and the later the maximum tolerance is expressed. However, from these studies it is not clear whether it is the level of heat damage after preheating or the preheating temperature itself which is most important for thermotolerance. A recent *in vitro* study (Nielsen & Overgaard, in press) showed the same degree and kinetics of thermotolerance irrespective of the preheating temperature if the preheating times were adjusted to

Correspondence to: Dr O. S. Nielsen, The Institute of Cancer Research, Radiumstationen, Nörrebrogade 44, DK-8000 Aarhus C, Denmark.

give identical survival levels. Similarly, in a study on mouse pinna (ears) *in vivo*, Law (1981) found no significant difference between either the degree or the kinetics of thermotolerance induced by preheating at temperatures between 41·5 and 45·5°C if the pretreatments were adjusted to give the same degree of ear necrosis. Unfortunately, a paucity of information exists about the dependence of thermotolerance on the priming heat dose in solid tumours *in vivo*. Recently, Urano and co-workers (Maher *et al.*, 1981; Urano *et al.*, 1982) have shown that the degree of thermotolerance in a solid tumour increased with prolonged preheating time at 45·5°C. However, as these studies were performed at a single temperature with only one fractionation interval (24 h), they did not provide information about the time course of thermotolerance in solid tumours.

In the present study, the importance of the primary heat treatment temperature and heating time for the degree and kinetics of thermotolerance was investigated in a solid mammary carcinoma. The investigations were based on an experimental tumour model, which we have recently established for quantitative studies on the development of thermotolerance in solid tumours (Kamura *et al.*, 1982). In addition, studies were performed to determine the relationship between heat effect and temperature on tumours given single heat treatment.

### MATERIALS AND METHODS

*Animal tumour system.*—Ten-to-12-week-old male and female C3D2F1/Bom (C3H/Tif ♀ × BDA/2 ♂) mice were challenged with a spontaneously C3H/Tif mammary carcinoma, which was propagated by serial transplantation. Tumour material for inoculation was obtained by sterile dissection of large flank tumours. Macroscopically viable tumour tissue was minced with a pair of scissors, and 5–10 $\mu$l of this minced tumour was injected into the foot on the right hind limb of the experimental animals. The transplant take was 95%. Tumours reaching a volume of $\sim$ 200 mm$^3$ (determined by the $\pi/6 \times D1 \times D2 \times D3$ formula in which the D's are 3 orthogonal diameters) within 12–24 days after inoculation were used for treatment.

*Hyperthermic treatment.*—The mice were randomly allocated into the different treatment groups. All treatments were administered to unanaesthetized mice placed in lucite jigs with the tumour-bearing legs loosely fixed with tape without impairing the blood flow to the feet (Overgaard, 1981). Local hyperthermia was performed with the tumour-bearing leg immersed into a circulating water bath stabilized to $\pm$0·05°C of the adjusted temperature. The intratumoral temperature stabilized within a few minutes to approximately 0·2°C below the water-bath temperature. The water bath was therefore adjusted to 0·2°C above the desired tumour temperature, and all temperatures mentioned in this paper refer to the intratumoral temperature. Further details of the temperature measurements and the treatment procedures are described elsewhere (Overgaard, 1980a, b).

*Evaluation of results.*—After treatment, tumour volume was measured daily. The tumour response was evaluated as tumour growth time, i.e. the time required for a tumour to reach a volume 5 times that of the first treatment day. As previously described (Kamura *et al.*, 1982), at a given temperature tumour growth time depends only on heating time but is independent of sex, batch of mice, and initial tumour volume (within 150–257 mm$^3$). Dose–response curves for tumour growth versus heating time were plotted by means of linear regression calculations. Student's *t*-test or analysis of variances were used for statistical analysis.

### RESULTS

### *Single heating*

The effect of a single heat treatment at 41·5–44·5°C for various periods is shown in Fig. 1. Tumour growth time was dependent on both temperature and heating time, and at all temperatures, there was a linear dose–response relationship between tumour growth time and heating time. Table I shows the calculated characteristics of these dose–response curves. The heat sensitivity, based on the slope value, gradually increased with higher temperatures, whereas the calculated intercept

FIG. 1.—Dose–response curves for solid tumours exposed to a single heat treatment at 41·5–44·5°C. All curves were plotted by means of linear regression calculations based on the individual mouse tumour growth times (calculated slope values, all significantly different from 0 ($P<0·001$)). Each point represents the mean of a group of mice (at least 5), the vertical bars representing $\pm 1$ s.e. See Table I for curve characteristics.

FIG. 2.—Arrhenius plot of tumours heated once only. Based on the dose–response curves in Fig. 1. The activation energies $\mu$ were calculated by linear regression analysis for the temperature ranges 41·5–42·5 and 42·5–44·5°C, respectively (calculated slope values, both significantly different from 0 ($P < 0·05$)). Vertical bars represent $\pm 1$ s.e.

TABLE I.—*Dose–response curve characteristics of tumours receiving a single heat treatment (from Fig.* 1)

| Temperature (°C) | No. of mice | Intercept[a] (days) | Slope[b] (/min) |
|---|---|---|---|
| 41·5 | 36 | 6·0 (5·8–6·2)[c] | 0·011 (0·008–0·014) |
| 42·0 | 54 | 6·0 (5·6–6·4) | 0·030 (0·026–0·034) |
| 42·5 | 69 | 6·0 (5·7–6·3) | 0·070 (0·065–0·075) |
| 43·0 | 71 | 6·0 (5·7–6·3) | 0·089 (0·084–0·094) |
| 43·5 | 149 | 5·9 (5·5–6·3) | 0·147 (0·138–1·156) |
| 44·5 | 50 | 6·0 (5·6–6·4) | 0·308 (0·284–0·332) |

[a] Not significantly different from the tumour growth time for untreated controls = 6·0 days (5·8–6·2).
[b] All values are significantly different from 0 ($P<0·001$).
[c] 95% confidence limits in parentheses.

values of the curves did not differ significantly from the observed control tumour growth time.

To analyse the relationship between heat inactivation and temperature, the slope values from Table I were plotted as a function of the reciprocal temperature (Fig. 2). The slope of the resulting Arrhenius plot is a measure of the activation energy $\mu$. This Arrhenius plot showed an infection point at 42·5°C. The calculated activation energy was 635 kJ/mol (152kcal/mol) and 1508kJ/mol (361 kcal/mol), respectively, above and below the inflection point. For the remainder of our studies heating was only performed at temperatures above the inflection point (i.e. $\geq$ 42·5°C).

### *Effect of preheating temperature on thermotolerance*

Based on the Arrhenius curve, the heating times at 42·5 and 44·5°C, resulting in the same tumour growth time as that of 43·5°C for 30 min, were calculated. Experiments were then made to determine the dependence of thermotolerance on preheating temperature when the heating times were adjusted to give the same level of heat damage.

Recovery from hyperthermic damage at

FIG. 3.—Tumour growth time of solid tumours treated with 2 heat treatments separated by different intervals. The first treatment was for 60 min at 42·5°C (▲), 30 min at 43·5°C (●) and 15 min at 44·5°C (○) respectively, followed by a second treatment for 60 min at 43·5°C. Each point represents the mean of a group of mice (at least 5), the vertical bars representing $\pm 1$ s.e.

various temperatures was evaluated by application of 2 separate hyperthermic treatments at different intervals. The first heating was for 60 min at 42·5°C, 30 min at 43·5°C and 15 min at 44·5°C, respectively, the second for 60 min at 43·5°C, respectively, the second for 60 min at 43·5°C (Fig. 3). To ensure that the warm-up time was the same for all treatment groups, irrespective of pretreatment temperature, the 2 treatments were separated by 5 min at the 0-h interval. At all 3 temperatures the tumour growth time decreased with increasing interval to reach its minimum at a 16-h interval, and there was no marked difference between the 3 recovery curves. These recovery curves may illustrate the kinetics of thermotolerance, and thus, the data in Fig. 3 may indicate that thermotolerance developed identically at all 3 temperatures with a maximum at a 16-h interval. This was determined quantitatively at the time of maximum recovery by giving graded second doses at 43·5°C (Fig. 4). Thermotolerance developed, as demonstrated by a lesser slope of the curves for tumours preheated at a 16-h interval as compared to that for tumours treated at a 0-h interval. Table II shows the dose–response curve characteristics and the values of the "thermotolerance ratio" (TTR). As previously discussed (Kamura *et al.*, 1982), this ratio is a measure of the degree of thermotolerance induced by a single hyperthermic treatment and developed during a postheating interval. There was no difference between the intercept and $TTR_{16}$ values at the 3 temperatures. So, for a given level of heat damage, the degree of the subsequent thermotolerance was almost independent of temperature, i.e. the relationship between heating time and temperature for the induction of thermotolerance was the same as that found for cell killing by hyperthermia. Therefore, the experiments on the influence of heating time were carried out at the same temperature, i.e. 43·5°C.

*The effect on thermotolerance of varying the preheating time at* 43·5°*C*

The kinetics of thermotolerance as a function of preheating time is illustrated by the recovery curves in Fig. 5 which shows the tumour response to 43·5°C for 60 min at various intervals following preheating at 43·5°C for 3·5, 7·5, 15, 30, or 45 min, respectively. It is seen that the longer the preheating time, the longer the fraction interval necessary to obtain maximum thermotolerance and the longer the time for complete decay of thermotolerance. A quantitative evaluation of the development of thermotolerance at the time of maximum recovery (Fig. 6 and Table III) showed also that the degree of thermotolerance clearly depended on the duration of the priming 43·5°C-treatment. The longer the preheating time at 43·5°C, the higher the maximum thermotolerance ratio ($TTR_{max}$).

This dependence on the primary heating time is seen clearly in Figs 7 and 8, which show that both $TTR_{max}$ (Fig. 7) and the interval required to obtain $TTR_{max}$ (Fig. 8) were linear functions of the preheating time.

TABLE II.—*Dose–response curve characteristics of tumours heated at 43·5°C 16 h after preheating at different temperatures*

| Preheat | | | | Heat treatment at 43·5°C | | | | |
|---|---|---|---|---|---|---|---|---|
| | | | | 0 h after preheat | | 16 h after preheat | | |
| Temperature (°C) | Heating time (min) | No. of mice | Tumour growth time after preheat (days) | Intercept[a] (days) | slope (/min) | Intercept[a] (days) | slope (/min) | $TTR_{16}$[b] |
| 42·5 | 60 | 139 | 9·9 (9·3–10·5)[c] | 10·0 (9·1–10·9) | 0·154 (0·134–0·174) | 9·9 (9·3–10·5) | 0·030 (0·025–0·035) | 5·1 (4·8–5·4) |
| 43·5 | 30 | 152 | 10·2 (9·6–10·8) | 10·3 (9·7–11·0) | 0·145 (0·135–0·155) | 10·2 (9·6–10·8) | 0·028 (0·020–0·036) | 5·2 (4·2–6·2) |
| 44·5 | 15 | 93 | 10·3 (9·1–11·5) | 10·0 (8·9–11·1) | 0·157 (0·131–0·171) | 9·7 (8·9–10·5) | 0·029 (0·021–0·037) | 5·4 (4·4–6·4) |

[a] Not significantly different from the tumour growth time after preheat ($P > 0·60$).

[b] Thermotolerance ratio (TTR) = slope (0h interval)/slope (16h interval); no significant difference ($P > 0·60$).

[c] 95% confidence limits in parentheses.

FIG. 4.—Development of thermotolerance in solid tumours treated at 43·5°C 16 h after preheating for 60 min at 42·5°C (▲), 30 min at 43·5°C (●) and 15 min at 44·5°C (○) respectively. Curves at 0 h represent tumours treated at 43·5°C at a 0h interval after preheating. Curves were plotted by linear regression calculations based on the individual mouse tumour growth times (calculated slope values, all significantly different from 0 ($P < 0{\cdot}001$)). Each point represents the mean of a group of mice (at least 5), the vertical bars representing ±1 s.e. See Table II for curve characteristics.

### DISCUSSION

The kinetics of thermal inactivation of most cell lines differ above and below 42·5–43·0°C, at indicated by an inflection on an Arrhenius curve (Dewey *et al.*, 1977; Bhuyan, 1979; Henle, 1982; Nielsen *et al.* (in press). In the present study, the Arrhenius curve (Fig. 2) also showed an inflection point at 42·5°C below which a 2–3-fold increase in the activation energy was observed. A similar biphasic pattern was also obtained in other studies of normal tissues and tumours heated *in vivo* (Overgaard & Suit, 1979; Henle, 1982). However, in these *in vivo* studies the measurement of heat effects was based on fixed end-points. The Arrhenius analysis requires the measurement of a rate under at least quasi-steady-state conditions, and thus these *in vivo* data cannot be represented on an Arrhenius plot without assuming that the accumulation of heat

FIG. 5.—Tumour growth time of solid tumours determined at various intervals after an initial exposure at 43·5°C for 3·5 (△), 7·5 (■), 15 (□), 30 (●), and 45 (◇) min, respectively. The second treatment was at 43·5°C for 60 min. The tumour growth time after preheating is indicated in Table III. Each point represents the mean of a group of mice (at least 5), the vertical bars representing ± 1 s.e.

FIG. 6.—Maximal thermotolerance at 43·5°C induced by preheating for different times at 43·5°C. The fractionation intervals represent the time intervals of maximum recovery obtained from Fig. 5. The curves are corrected for the effect of preheating, *i.e.* the increase in tumour growth time caused by the indicated preheating times is not included. The curve at 0 h represents single heat treatment at 43·5°C. All curves were plotted by linear regression calculations based on the individual tumour growth times (calculated slope values, all significantly different from 0 ($P < 0{\cdot}01$)). Each point represents the mean of a group of mice (at least 5), the vertical bars representing ±1 s.e. See Table III for curve characteristics.

FIG. 7.—Degree of maximal thermotolerance ($TTR_{max}$) as a function of the duration of the preheating time. The curve was fitted by linear regression analyses (calculated slope value differed significantly from 0 ($P < 0{\cdot}001$)). Actual values are given in Table III. Vertical bars represent ±1 s.e.

FIG. 8.—Fractionation interval required for the development of maximal thermotolerance ($TTR_{max}$) as a function of the duration of the primary heating time. The curve was fitted by linear regression analyses (calculated slope value differed significantly from 0 ($P < 0{\cdot}001$)). Actual values are given in Table III.

damage *in vivo* is a purely exponential function of heating time (Myers *et al.*, 1980; Henle in press). This assumption is unnecessary in the present study, since the use of tumour growth time as an end-point provided a graded quantitative response to hyperthermia. It should be noted that this assay of tumour response was based on the existence of a linear relationship between tumour growth time and heating time (Fig. 1), and on the fact that the regrowth rate of tumours subjected to hyperthermia did not differ from that of untreated tumours (Kamura *et al.*, 1982). The significance of this independence of the regrowth rate on heating time has recently been discussed by Wheldon & Hingston (1982).

The present experiments showed that thermotolerance could be induced by a prior heating at temperatures ranging from 42·5 to 44·5°C, and the relationship between heating time and temperature for this induced thermotolerance was the same as that found for cell killing by hyperthermia. In other words, both the degree and kinetics of thermotolerance were independent of the preheating tem-

TABLE III.—*Dose–response characteristics of tumours treated at 43·5°C at the time interval of maximum recovery[a] after preheating for different times at 43·5°C*

| Preheat at 43·5°C (min) | No. of mice | Tumour growth time after preheat (days) | Time[a] interval of $TTR_{max}$ (h) | Intercept[b] (days) | Slope (/min) | $TTR_{max}$[c] |
|---|---|---|---|---|---|---|
| 0 | 149 | — | — | 5·9 (5·5–6·3) | 0·147 (0·138–0·156) | 1·0 |
| 3·5 | 47 | 6·0 (5·6–6·4)[d] | 2 | 5·7 (5·0–6·4) | 0·094 (0·083–0·105) | 1·6 (1·3–1·9) |
| 7·5 | 74 | 6·4 (6·0–6·8) | 4 | 6·1 (5·4–6·8) | 0·066 (0·059–0·073) | 2·2 (1·9–2·5) |
| 15 | 59 | 7·5 (6·9–8·1) | 8 | 7·1 (6·5–7·7) | 0·040 (0·033–0·047) | 3·7 (3·3–4·1) |
| 30 | 91 | 10·2 (9·6–10·8) | 16 | 10·2 (9·9–10·5) | 0·028 (0·022–0·034) | 5·2 (4·2–6·2) |
| 45 | 80 | 12·8 (12·2–13·4) | 28 | 12·2 (11·4–13·0) | 0·019 (0·013–0·024) | 7·7 (6·6–8·8) |

[a] Obtained from Fig. 5.
[b] Not significantly different from the tumour growth after preheat ($P > 0·60$).
[c] Maximum thermotolerance ratio ($TTR_{max}$) = slope (no preheat)/slope (after preheat).
[d] 95% confidence limits in parentheses.

perature (in the range 42·5–44·5°C) if the heating times were adjusted to give the same degree of heat damage. This agrees with data from L1A2 cells *in vitro* (Nielsen & Overgaard, in press) and mouse pinna (ears) *in vivo* (Law, 1981).

On the other hand, at a given temperature the development of thermotolerance in the tumours clearly depended on the duration of the primary heat treatment (Figs 5 and 6). Both the fractionation interval necessary to obtain maximum thermotolerance increased as the preheating time was increased. This agrees with earlier observations on cell cultures *in vitro* (Gerner *et al.*, 1976; Henle *et al.*, 1978; Li *et al.*, 1982; Nielsen & Overgaard, in press, and on different normal tissues *in vivo* (Law *et al.*, 1979; Hume & Marigold, 1980; Urano *et al.*, 1980; Rice *et al.*, 1982; Field & Anderson, in press). Recently, studies on solid tumours have also shown that at a given interval the degree of thermotolerance increased as the preheating time was increased (Maher *et al.*, 1981; Urano *et al.*, 1982). However, these studies did not provide information about the time course of thermotolerance.

It has been demonstrated on cell cultures (Henle *et al.*, 1978; Li & Hahn, 1980; Li *et al.*, 1982; Nielsen & Overgaard, in press) that the rate of both development and decay of thermotolerance are independent of preheating time. The data in Fig. 5 may also indicate that the rate of decay was independent of the preheating time, although these data provide less detailed information on the decay rate than the *in vivo* studies. In contrast, the rate of development seemed to be faster following short pretreatments (Fig. 5), as also suggested by Urano *et al.* (1982). In addition, a recent *in vitro* study (Nielsen & Overgaard, in press) has shown that preheating also induces a delay in onset of thermotolerance, and that this lag period increases with longer priming treatment periods. Such a delay period was not observed in the present study.

The time for loss of thermotolerance clearly depended on the preheating time as also demonstrated on normal tissues *in vivo* (Law *et al.*, 1979; Hume & Marigold, 1980). After preheating for 15 min or longer this time for loss of thermotolerance coincided with the occurrence of renewed tumour growth after prior heating. With shorter pretreatments the tumour regrowth was observed before the time for complete decay of thermotolerance. However, as this regrowth appeared late in the decay period, it may not have influenced the results.

Both the $TTR_{max}$ and the time interval required for its development were linear functions of the priming treatment time (Figs 7 and 8). A similar relationship has also been demonstrated for cell cultures (Henle *et al.*, 1979; Nielsen & Overgaard, in press). However, in these studies the $TTR_{max}$ and the time required to obtain $TTR_{max}$ were also linear functions of the logarithm of the relative survival following preheating. Also the data from Law *et al.* (1979) may indicate a linear relationship between the maximum degree of thermotolerance in mouse ears and the duration of the prior heat treatment at 43·5°C, at least after pretreatments up to 20 min. With pretreatments longer than 20 min, the maximum degree of thermotolerance did not increase further. Such a plateau was not observed in the present study which may be due to a difference in the experimental design. However, in concordance with the present studies, Law *et al.* (1979) observed that even pretreatments as brief as a few minutes at 43·5°C, which had no detectable heat effects *per se*, induced thermotolerance.

If the observation of the degree and the kinetics of thermotolerance as linear functions of the level of heat damage following preheating is a general phenomenon, it would have clinical implications. As previously discussed in detail (Nielsen & Overgaard, in press), the degree and kinetics of thermotolerance in different tissues induced by equal pretreatments show great variation and therefore information on thermotolerance in one tissue may not predict the degree and kinetics of tolerance in others. Despite this variation, if the tumour suffers greater primary heat damage than the normal tissues, the tumour may develop greater thermal resistance to a subsequent treatment than the normal tissues, thus cancelling any therapeutic gain. On the other hand, this will depend on the heat sensitivities of the 2 tissues and on the interval between the treatments. This may be further complicated by heterogeneous tumour heating. Due to either vascular cooling or a technically heterogeneous heat distribution, the development of thermotolerance in one part of a tumour may differ from that of other areas within the same tumour. So, given these complications, the problems related to the development of thermotolerance may pose such difficulties that clinical hyperthermia should be administered with sufficiently long fractionation intervals to ensure complete disappearance of thermotolerance (Nielsen & Overgaard, in press; Urano *et al.*, 1982).

In conclusion, the present data indicate that in the temperature range 42·5–44·5°C, both the degree and kinetics of thermotolerance in a solid tumour depend on the level of heat damage following preheating irrespectively of the treatment temperature and heating time used to obtain this level of heat damage.

We wish to thank I. M. Johansen and L. Baltersen for enthusiastic and skilful technical help; L. Wagner and E. B. Mathiesen for secretarial assistance; A. H. Andersen, Institute of Theoretical Statistics, University of Aarhus, for providing us with computer programs and valuable help with the statistical analyses.

This work was supported by the Danish Cancer Society (grants 24/79 and 87/79), and Ingeborg and Leo Danin's Foundation for Scientific Research.

### REFERENCES

BHUYAN, B. K. (1979) Kinetics of cell kill by hyperthermia. *Cancer Res.*, **39**, 2277.

DEWEY, W. C., HOPWOOD, L. E., SAPARETO, S. A. & GERWECK, L. E. (1977) Cellular responses to combinations of hyperthermia and radiation. *Radiology*, **123**, 463.

FIELD, S. B. & ANDERSON, R. L. (1982) Thermotolerance: a review of observations and possible mechanisms (in press).

GERNER, E. W., BOONE, R., CONNOR, W. G., HICKS, J. A. & BOONE, M. L. M. (1976) A transient thermotolerant survival response produced by single thermal doses in HeLa cells. *Cancer Res.*, **36**, 1035.

HENLE, K. J. (1982) Arrhenius analysis of thermal responses. In *Hyperthermia in cancer therapy* (Ed. Storm *et al.*). Boston: Hall & Co. (in press).

HENLE, K. J. & DETHLEFSEN, L. A. (1978) Heat fractionation and thermotolerance: a review. *Cancer Res.*, **38**, 1843.

HENLE, K. J., BITNER, A. F. & DETHLEFSEN, L. A. (1979) Induction of thermotolerance by multiple heat fractions in Chinese hamster ovary cells. *Cancer Res.*, **39**, 2486.

Henle, K. J., Karamuz, J. E. & Leeper, D. B. (1978) Induction of thermotolerance in Chinese hamster ovary cells by high (45°) or low (40°) hyperthermia. *Cancer Res.*, **38**, 570.

Hume, S. P. & Marigold, J. C. L. (1980) Transient, heat-induced thermal resistance in the small intestine of mouse. *Radiat. Res.*, **82**, 526.

Kamura, T., Nielsen, O. S., Overgaard, J. & Andersen, A. H. (1982) Development of thermotolerance during fractionated hyperthermia in a solid tumour *in vivo*. *Cancer Res.*, **42**, 1744.

Law, M. P. (1981) The induction of thermal resistance in the ear of the mouse by heating at temperatures ranging from 41·5 to 45·5°C. *Radiat. Res.*, **85**, 126.

Law, M. P., Coultas, P. G. & Field, S. B. (1979) Induced thermal resistance in the mouse ear. *Br. J. Radiol.*, **52**, 308.

Li, G. C. & Hahn, G. M. (1980) A proposed operational model of thermotolerance based on effects of nutrients and the initial treatment temperature. *Cancer Res.*, **40**, 4501.

Li, G. C., Fisher, G. A. & Hahn, G. M. (1982) Induction of thermotolerance and evidence for a well-defined thermotropic cooperative process. *Radiat. Res.*, **89**, 361.

Maher, J., Urano, M., Rice, L. & Suit, H. D. (1981) Thermal resistance in a spontaneous murine tumour. *Br. J. Radiol.*, **54**, 1086.

Myers, R., Robinson, J. E. & Field, S. B. (1980) The relationship between heating time and temperature for inhibition of growth in baby rat cartilage by combined hyperthermia and x-rays. *Int. J. Radiat. Biol.*, **38**, 373.

Nielsen, O. S. & Overgaard, J. (1979) Effect of extracellular pH on thermotolerance and recovery of hyperthermic damage *in vitro*. *Cancer Res.*, **39**, 2772.

Nielsen, O. S. & Overgaard, J. (1982) Influence of time and temperature on the kinetics of thermotolerance in L1A2 cells *in vitro*. *Cancer Res.* (in press).

Nielsen, O. S., Henle, K. J. & Overgaard, J. (1982) Arrhenius analysis of survival curves from thermotolerant and step-down heated L1A2 cells *in vitro*. *Radiat. Res.* (in press).

Overgaard, J. (1980*a*) Simultaneous and sequential hyperthermia and radiation treatment of an experimental tumor and its surrounding normal tissue *in vivo*. *Int. J. Radiat. Oncol. Biol. Phys.*, **6**, 1507.

Overgaard, J. (1980*b*) Effect of misonidazole and hyperthermia on the radiosensitivity of a C3H mouse mammary carcinoma and its surrounding normal tissue. *Br. J. Cancer*, **41**, 10.

Overgaard, J. (1981) Effect of hyperthermia on the hypoxic fraction in an experimental mammary carcinoma *in vivo*. *Br. J. Radiol.*, **54**, 245.

Overgaard, J. & Suit, H. D. (1979) Time–temperature relationship in hyperthermic treatment of malignant and normal tissue *in vivo*. *Cancer Res.*, **39**, 3248.

Rice, L. C., Urano, M. & Maher, J. (1982) The kinetics of thermotolerance in the mouse foot. *Radiat. Res.*, **89**, 291.

Spiro, I. J., Sapareto, S. A., Raaphorst, G. P. & Dewey, W. C. (1982) The effect of chronic and acute heat conditioning on the development of thermal tolerance. *Int. J. Radiat. Oncol. Biol. Phys.*, **8**, 53.

Urano, M., Rice, L. C. & Montoya, V. (1982) Studies on fractionated hyperthermia in experimental animal systems. II. Response of murine tumors to two or more doses. *Int. J. Radiat. Oncol. Biol. Phys.*, **8**, 227.

Urano, M., Rice, L., Kahn, J. & Sedlacek, R. S. (1980) Studies on fractionated hyperthermia in experimental animal systems. I. The foot reaction after equal doses: heat resistance and repopulation.

Wheldon, T. E. & Hingston, E. C. (1982) Differential effect of hyperthermia and x-irradiation on regrowth rate and tumour-bed effect for a rat sarcoma. *Br. J. Cancer*, **45**, 265.