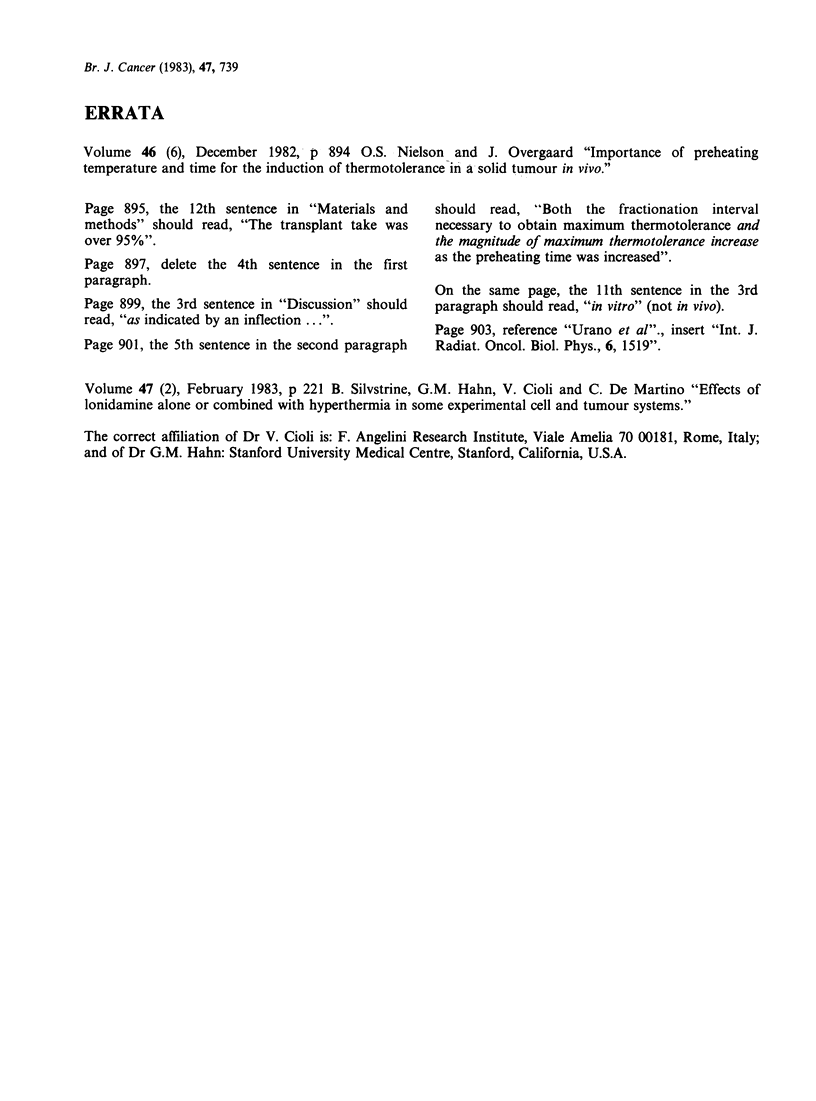# Errata

**Published:** 1983-05

**Authors:** 


*Br. J. Cancer* (1983), **47**, 739

### ERRATA

Volume **46** (6), December 1982, p 894 O.S. Nielson and J. Overgaard "Importance of preheating temperature and time for the induction of thermotolerance in a solid tumour *in vivo*."

Page 895, the 12th sentence in "Materials and methods" should read, "The transplant take was over 95%".

Page 897, delete the 4th sentence in the first paragraph.

Page 899, the 3rd sentence in "Discussion" should read, "*as* indicated by an inflection ...".

Page 901, the 5th sentence in the second paragraph should read, "Both the fractionation interval necessary to obtain maximum thermotolerance *and the magnitude of maximum thermotolerance increase* as the preheating time was increased".

On the same page, the 11th sentence in the 3rd paragraph should read, "*in vitro*" (not *in vivo*).

Page 903, reference "Urano *et al*"., insert "Int. J. Radiat. Oncol. Biol. Phys., **6**, 1519".